# Predictors of Thoracic Complications After Bilateral Diaphragmatic Stripping During Cytoreductive Surgery for Advanced Ovarian Cancer

**DOI:** 10.3390/medicina62050818

**Published:** 2026-04-25

**Authors:** Carlo Ronsini, Federica Anzelmo Sciarra, Giuseppe Cucinella, Mariano Catello Di Donna, Cono Scaffa, Mario Fordellone, Stefano Restaino, Manuela Ludovisi, Giuseppe Vizzielli, Vito Chiantera

**Affiliations:** 1 Unit of Gynecologic Oncology, National Cancer Institute, IRCCS, Fondazione “G. Pascale”, 80131 Naples, Italy; federicaanzelmosciarra@gmail.com (F.A.S.); giuseppecucinella@outlook.com (G.C.); mariano.didonna@istitutotumori.na.it (M.C.D.D.); c.scaffa@istitutotumori.na.it (C.S.); vito.chiantera@istitutotumori.na.it (V.C.); 2 Unit of Gynecology and Obstetrics, Department of Woman, Child and General and Specialized Surgery, University of Campania “Luigi Vanvitelli”, 80138 Naples, Italy; 3 Medical Statistics Unit, Department of Mental and Physical Health and Preventive Medicine, University of Campania “Luigi Vanvitelli”, 80138 Naples, Italy; mario.fardellone@unicampania.it; 4 Unit of Obstetrics and Gynecology, “Santa Maria della Misericordia” University Hospital, Azienda Sanitaria Universitaria Friuli Centrale, 33100 Udine, Italy; restaino.stefano@gmail.com (S.R.); giuseppevizzielli@yahoo.it (G.V.); 5 Department of Life, Health and Environmental Sciences, University of L’Aquila, 67100 L’Aquila, Italy; mludovisi@gmail.com

**Keywords:** ovarian cancer, cytoreductive surgery, diaphragmatic stripping, thoracic complications, BMI, ASA score

## Abstract

*Background and Objective*: This study aimed to identify preoperative and intraoperative factors associated with thoracic complications after bilateral diaphragmatic stripping during cytoreductive surgery for advanced ovarian cancer. *Materials and Methods*: A retrospective observational study was conducted at the Gynecologic Oncology Unit of the National Cancer Institute “G. Pascale”, Naples, Italy. We included patients who underwent bilateral diaphragmatic stripping between July 2023 and October 2025. Demographic, surgical, and anesthesiologic parameters were recorded. Univariate logistic regression was performed, and a restricted multivariate model including only variables significant at univariate analysis was used to assess predictors of thoracic complications. *Results*: Forty-seven patients were analyzed, 10 (21%) of whom developed postoperative thoracic complications. Patients with thoracic complications had a higher body mass index (median 28.4 kg/m^2^, IQR 26.4–29.3 vs. 23.9 kg/m^2^, IQR 22.8–27.3; *p* = 0.003) and higher ASA scores (*p* = 0.033). In univariate analysis, ASA (odds ratio [OR] 3.90, 95% confidence interval [CI] 1.12–17.94, *p* = 0.046) and BMI (OR 1.45, 95% CI 1.14–2.02, *p* = 0.009) were significantly associated with thoracic complications. In multivariate analysis, only BMI remained an independent predictor (OR 1.599, 95% CI 1.13–2.68, *p* = 0.027). *Conclusions*: Elevated BMI was independently associated with an increased risk of thoracic complications after bilateral diaphragmatic stripping in cytoreductive surgery for ovarian cancer. Careful perioperative management and preventive strategies should be considered in overweight patients.

## 1. Introduction

Diaphragmatic involvement is common in advanced epithelial ovarian cancer, occurring in a substantial proportion of patients undergoing primary or interval cytoreductive surgery [1,2]. Achieving complete cytoreduction, including clearance of disease in the upper abdomen, remains a pivotal prognostic factor for overall survival [3,4]. Accordingly, diaphragmatic peritonectomy or stripping is frequently required to remove tumor implants from the diaphragmatic surface [5].

Despite clear oncologic benefits, diaphragmatic stripping is associated with postoperative thoracic complications such as pleural effusion, pneumothorax, and pulmonary infection [6,7]. Reported rates range from 10% to 35% and may depend on surgical laterality, technique, and perioperative management [8,9]. Patient-related factors and operative choices, such as chest drain placement, may modulate the risk of these events [7,10,11]. However, evidence focusing specifically on characteristics of patients undergoing bilateral diaphragmatic stripping is limited.

This study aimed to identify perioperative factors associated with thoracic complications in patients undergoing bilateral diaphragmatic stripping during cytoreductive surgery for advanced ovarian cancer. Moreover, bilateral diaphragmatic stripping represents a specific surgical subset characterized by extensive upper abdominal disease, which may limit the applicability of findings derived from broader cytoreductive surgery populations.

### Objective

The objective of this study was to identify clinical and perioperative predictors of thoracic complications in patients undergoing bilateral diaphragmatic stripping as part of cytoreductive surgery for advanced epithelial ovarian cancer.

The rationale stems from the observation that, while diaphragmatic peritonectomy is essential to achieve optimal cytoreduction and improve survival, it entails a non-negligible risk of postoperative respiratory morbidity. To clarify these aspects, we conducted a retrospective analysis of consecutive patients treated at a single tertiary cancer center, aiming to quantify the incidence of thoracic complications and to assess which perioperative factors may independently predict their occurrence.

## 2. Materials and Methods

### 2.1. Study Design

We conducted a retrospective observational study at the Gynecologic Oncology Unit of the National Cancer Institute, IRCCS, Fondazione “G. Pascale”, Naples, Italy. The study included consecutive patients who underwent bilateral diaphragmatic stripping between July 2023 and October 2025, representing the complete accrual period. All data were fully collected and available at the time of analysis. The study followed the STROBE statement for observational studies [12]. In accordance with Italian regulations for retrospective studies and the principles of the Declaration of Helsinki, formal Institutional Review Board approval was not required. All patients routinely sign informed consent for the anonymous use of their clinical data for research purposes at the time of treatment.

### 2.2. Setting

All surgeries were performed by the same dedicated gynecologic oncology team, with the intent of complete cytoreduction. Data were collected from prospectively maintained institutional databases and electronic medical records.

### 2.3. Participants

Inclusion criteria were: (1) histologically confirmed epithelial ovarian cancer; (2) bilateral diaphragmatic stripping as part of cytoreductive surgery; (3) complete clinical, surgical, and anesthesiologic data. Exclusion criteria were: (1) unilateral diaphragmatic stripping; (2) full-thickness diaphragmatic resection or evidence of diaphragmatic perforation during surgery; (3) incomplete data about surgery; (4) use of HIPEC during surgery; (5) postoperative follow-up shorter than 30 days. Placement of an intraoperative thoracic drain was left to the surgeon’s discretion. All included patients underwent bilateral diaphragmatic stripping, representing a homogeneous surgical subgroup. Cases involving full-thickness diaphragmatic resection were excluded according to predefined criteria.

### 2.4. Variables

We collected age (years), BMI (kg/m^2^), ASA score (modeled as an ordinal variable assuming a monotonic increase in perioperative risk), Charlson comorbidity index, type of diagnosis (primary vs. recurrent), surgical approach (Primary Debulking Surgery, PDS, vs. Interval Debulking Surgery, IDS), operative time (minutes), estimated blood loss (mL), Aletti’s surgical complexity score, and number of preoperative chemotherapy cycles when performed. While three to four cycles are typically considered standard neoadjuvant chemotherapy, a subset of patients received extended preoperative chemotherapy due to clinical or organizational factors. Therefore, cycles beyond four should be interpreted as prolonged preoperative treatment rather than standard neoadjuvant therapy. BMI categories were defined according to the World Health Organization classification. Preoperative clinical status was evaluated using both the ASA score, reflecting global anesthesiologic risk and physiological reserve, and the Charlson Comorbidity Index, which quantifies the burden of comorbid conditions. The presence and volume of ascites at the time of surgery, although clinically relevant in advanced ovarian cancer, were not systematically quantified in the institutional database and therefore could not be included as covariates. The “primary” diagnosis referred to patients undergoing cytoreductive surgery at the initial presentation of advanced epithelial ovarian cancer, whereas “recurrent” indicated those operated for disease relapse after a previous complete response. The Peritoneal Cancer Index (PCI) was not systematically available in the institutional dataset. However, the requirement for bilateral diaphragmatic stripping can be considered a surrogate marker of extensive upper abdominal disease and advanced peritoneal dissemination. Neoadjuvant chemotherapy, when administered, consisted of carboplatin (AUC 5) combined with paclitaxel according to institutional standards before interval debulking surgery. The American Society of Anesthesiologists (ASA) Physical Status Classification System was used to evaluate the patients’ preoperative anesthesiologic risk, ranging from 1 (healthy patient) to 5 (moribund patient not expected to survive without surgery). Aletti’s Surgical Complexity Score quantified the extent of surgical effort based on the number and type of upper abdominal procedures performed during cytoreduction, providing an objective measure of operative difficulty and tumor burden. Intraoperative fluid administration followed a standardized institutional anesthesiology protocol (goal-directed and weight-adjusted), aiming to maintain hemodynamic stability and urine output, with preferential use of balanced crystalloids. Therefore, fluid management was not treated as an independent variable. Thoracic complications included postoperative pleural effusion, pneumothorax, or pulmonary infection confirmed radiologically or clinically within 30 days after surgery. Only clinically relevant complications were considered, defined as: pleural effusion requiring drainage or thoracentesis; pneumothorax requiring chest tube placement; and pulmonary infection requiring antibiotic therapy. Ascites is a known determinant of postoperative pleural effusion due to transdiaphragmatic fluid migration and may represent a relevant confounder. However, it was not systematically quantified in the institutional dataset and therefore could not be included in the analysis. No reliable proxy variables, such as preoperative albumin or intraoperative fluid balance, were consistently available for inclusion.

### 2.5. Surgery and Outcomes of Interest

Bilateral diaphragmatic stripping was performed by sharp and blunt peritonectomy with extensive removal of the diaphragmatic peritoneum on both hemidiaphragms, aiming at complete macroscopic clearance of tumor involvement. The procedure was conducted according to intraoperative tumor distribution within a standardized surgical approach ensuring a meticulous hemostasis and systematic inspection of the pleural space. Energy devices were selected according to the intraoperative evaluation and the surgeon’s preference, and included monopolar or bipolar electrosurgery, advanced bipolar devices, or radiofrequency instruments. The choice of technology was made by the surgical team based on the extent of peritoneal involvement and proximity to critical structures, to achieve optimal tissue dissection and hemostasis while minimizing thermal injury. The placement of intraoperative thoracic drainage was based on the surgeon’s intraoperative assessment, including the extent of diaphragmatic dissection, the suspicion of pleural opening, and intraoperative evaluation of air leakage. Therefore, not all patients undergoing diaphragmatic stripping received prophylactic drainage. Postoperative management included monitoring of output and radiologic evaluation, with removal typically performed when drainage output was minimal and no residual pleural collection was observed. Greater omentectomy was systematically performed as a radical omentectomy in all patients, in accordance with established surgical standards for advanced ovarian cancer, aiming at complete macroscopic cytoreduction.

### 2.6. Statistical Analysis

Given the limited sample size, normal distribution of continuous variables could not be assumed. Therefore, continuous variables were summarized as median and interquartile range (IQR, 25th–75th percentile). Between-group comparisons were performed using the Wilcoxon rank-sum test for continuous variables and Fisher’s exact test for categorical variables [13,14,15]. Predictors of thoracic complications were evaluated using univariate logistic regression. Given the limited number of events, a parsimonious and clinically driven analytical strategy was adopted. Only variables showing statistically significant differences between groups and/or at univariate analysis were considered for multivariate modeling. Although multiple univariate models could be performed, their systematic use in this context may be methodologically questionable due to the increased risk of unstable estimates and type I error related to multiple testing. Therefore, univariate analyses were interpreted in an exploratory manner. Odds ratios (OR) and 95% confidence intervals (CI) were reported, with statistical significance set at *p* < 0.05. Analyses were performed in R (version 4.3.1). Given the exploratory nature of the study and the limited sample size, no a priori sample size calculation was performed. Therefore, the results should be interpreted as hypothesis-generating. Due to the limited number of events, the results of the multivariate analysis should be interpreted with caution.

### 2.7. Risk of Bias

To mitigate confounding, variables showing clinical plausibility and statistical significance at univariate analysis were included in the multivariate model, taking into account the limited number of events. Data extraction and analyses were independently reviewed by two investigators (CR and MF).

## 3. Results

A total of 47 patients met the inclusion criteria. Ten patients (21%) experienced postoperative thoracic complications, whereas 37 (79%) did not. Age was similar between groups (median 57 years, IQR 50–63 vs. 58 years, IQR 51–65; *p* = 0.9). Patients with thoracic complications had a significantly higher BMI (median 28.4 kg/m^2^, IQR 26.4–29.3 vs. 23.9 kg/m^2^, IQR 22.8–27.3; *p* = 0.003) and higher ASA scores (*p* = 0.033). Other variables, including operative time, estimated blood loss, and surgical complexity score, were not significantly different (all *p* > 0.3). No differences were observed according to the surgical approach (PDS vs. IDS) or the intraoperative placement of thoracic drainage. Baseline characteristics are summarized in Table 1.

### 3.1. Multivariate Analysis

**Figure 1:**
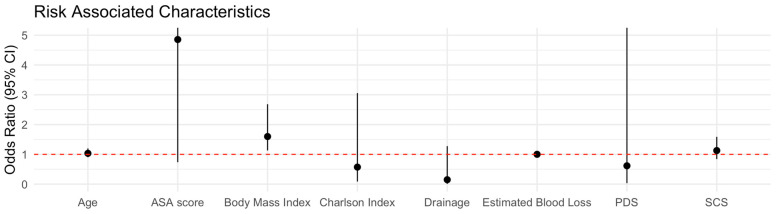
Forest Plot.

In univariate logistic regression, both ASA and BMI were significantly associated with thoracic complications: ASA (OR 3.90, 95% CI 1.12–17.94, *p* = 0.046) and BMI (OR 1.45, 95% CI 1.14–2.02, *p* = 0.009). In the multivariate model including only variables significant at univariate analysis (ASA and BMI), only BMI remained independently significant (OR 1.41, 95% CI 1.09–1.98, *p* = 0.021), whereas ASA was not significant. These findings indicate that increasing BMI is independently associated with postoperative thoracic complications after bilateral diaphragmatic stripping (Table 2, Figure 1).

### 3.2. Obesity

Overall, thoracic complications occurred in 21% of patients. The frequency increased across BMI categories: 8% in normal weight, 33% in overweight, and 50% in obese patients (*p* = 0.033) (Table 3).

The characteristics of patients who developed thoracic complications and the type of complication are shown in Table 4.

## 4. Discussion

### 4.1. Interpretation of Results

Higher BMI was independently associated with thoracic complications after bilateral diaphragmatic stripping, while ASA lost significance in adjusted models. These data suggest that obesity-related physiologic changes may contribute to postoperative respiratory morbidity in this setting. Notably, the timing of surgery (primary or interval debulking) and intraoperative techniques, such as drainage placement, did not differ significantly between patients with and without thoracic complications, suggesting that procedural factors did not show a measurable association with postoperative outcomes in this cohort. Similarly, comorbidities, as measured by the Charlson Comorbidity Index, were not associated with the occurrence of thoracic complications, supporting the hypothesis that BMI may represent one of the factors associated with postoperative respiratory morbidity in this cohort. Although completeness of cytoreduction is a recognized determinant of overall postoperative morbidity, no association was observed with thoracic complications in this selected cohort.

### 4.2. Comparison with Existing Literature

Our observed incidence of thoracic complications (21%) aligns with previously reported ranges for diaphragmatic peritonectomy [7,8,9]. Prior studies have implicated BMI as a driver of postoperative morbidity during cytoreductive surgery [16,17]. Mechanistically, obesity reduces pulmonary compliance and functional residual capacity and increases intra-abdominal pressure, potentially facilitating pleural effusions and atelectasis [18]. In our series, the surgical approach, whether Primary Debulking Surgery (PDS) or Interval Debulking Surgery (IDS), did not significantly affect the rate of thoracic complications. This observation suggests that, in this cohort, postoperative respiratory morbidity did not differ according to the timing of cytoreductive surgery and may be influenced by patient-specific factors such as BMI. Although IDS is often associated with reduced operative time and intraoperative blood loss following neoadjuvant chemotherapy, our data did not demonstrate a lower incidence of thoracic events in this group. Similar findings have been reported in previous studies comparing PDS and IDS, where the postoperative morbidity profile, including respiratory complications, was found to be comparable between approaches [19,20]. Although IDS is typically performed after neoadjuvant chemotherapy, which can theoretically reduce tumor burden and surgical aggressiveness, no protective effect was observed on respiratory outcomes. Bilateral diaphragmatic involvement requiring peritonectomy represents a marker of advanced upper abdominal disease and extensive peritoneal dissemination. Therefore, the present cohort reflects a highly selected population with substantial tumor burden. Although the Peritoneal Cancer Index (PCI) remains the standard tool for quantifying peritoneal spread, the uniform requirement for bilateral diaphragmatic stripping likely identifies patients within a similar range of advanced disease extent. These results suggest that the anatomical and technical complexity of bilateral diaphragmatic stripping likely contributes to the baseline risk of thoracic complications, with patient-related factors such as BMI potentially contributing to the individual risk of postoperative thoracic events. Ascites has also been recognized as a contributing factor to postoperative pleural effusion due to transdiaphragmatic fluid migration [6]. The inability to include ascites in the present analysis represents a relevant limitation and a potential source of residual confounding. Indeed, ascites may be associated with both disease burden and physiological status, potentially influencing the observed association between BMI and thoracic complications. The absence of this variable may have biased the results either toward or away from the null. Unfortunately, quantitative or even binary data on ascites were not consistently available, and no reliable surrogate markers were available in the current dataset.

### 4.3. Clinical Implications

Preoperative optimization of overweight and obese patients, careful intraoperative handling of the diaphragm, and selective pleural inspection may help reduce the risk of thoracic complications. Overall, the present findings support the need for a tailored surgical and perioperative approach. Diaphragmatic stripping plays a pivotal prognostic role in achieving complete cytoreduction and improving survival outcomes in advanced ovarian cancer. Consequently, the procedure should not be avoided solely due to the risk of thoracic morbidity. Instead, these risks must be anticipated, quantified, and managed proactively within a patient-centered framework.

Each patient should be fully informed about the realistic likelihood of thoracic complications based on their individual characteristics, including BMI, comorbidities, and surgical indication, to enable a transparent and shared decision-making process. The preoperative counseling phase thus becomes crucial, ensuring that patients understand both the oncologic benefit and the potential short-term respiratory risks of extensive upper abdominal surgery. Within the strategy of interval debulking surgery (IDS), the time frame between diagnosis and surgery should be regarded as an opportunity for multidisciplinary prehabilitation. Collaboration with a clinical nutritionist should be encouraged to promote targeted weight optimization, which may potentially contribute to reducing the risk of postoperative thoracic complications.

Furthermore, tailored perioperative respiratory physiotherapy, early ambulation, and individualized postoperative monitoring protocols can further enhance recovery and mitigate the respiratory burden associated with bilateral diaphragmatic stripping.

Ultimately, adopting a tailored approach that integrates nutritional, anesthesiologic, and surgical planning can allow surgeons to maintain aggressive oncologic intent while minimizing patient-specific risks, aligning the therapeutic strategy with the principles of precision surgery.

### 4.4. Strengths and Limitations

Strengths of this study include a homogeneous surgical approach, with all procedures performed by the same dedicated gynecologic oncology team using standardized operative techniques, and the use of multivariate modeling to control for potential confounders. Notably, patients who underwent hyperthermic intraperitoneal chemotherapy (HIPEC) were excluded, thereby eliminating a significant source of bias and physiological stress that could independently increase postoperative respiratory morbidity. This strengthens the internal validity of the findings and allows a more reliable assessment of factors related explicitly to diaphragmatic stripping. Nevertheless, several limitations should be acknowledged. The retrospective and single-center design inherently limits causal inference and generalizability. The sample size, while clinically informative, remains modest, limiting the power to detect associations for less frequent predictors. Furthermore, the absence of systematic long-term pulmonary follow-up prevents conclusions about the persistence or resolution of thoracic complications beyond the immediate postoperative period. Another relevant limitation concerns the regional characteristics of the study population. The cohort represents a Southern Italian population, where the prevalence of overweight and obesity is among the highest in Europe. This epidemiologic context may have amplified the impact of BMI on postoperative outcomes and might limit the external applicability of these results to populations with different baseline anthropometric distributions. Moreover, concomitant splenectomy was not evaluated as an independent predictor, which may represent an additional source of residual confounding. Future multicenter studies with larger and ethnically diverse samples are warranted to validate these findings, explore the impact of long-term pulmonary recovery, and assess the interaction between BMI, metabolic profile, and postoperative complications in advanced ovarian cancer surgery. Moreover, the absence of key clinical variables such as ascites or detailed pulmonary function assessment may have influenced the observed associations. Finally, the observational design of the study does not allow causal inference, and the findings should be interpreted as associative rather than causal. Furthermore, the limited number of events constrained the number of variables that could be included in the multivariate model, resulting in a suboptimal events-per-variable ratio. This may have increased the risk of model overfitting and limits the robustness of the adjusted estimates.

## 5. Conclusions

In patients undergoing bilateral diaphragmatic stripping during cytoreductive surgery for advanced ovarian cancer, elevated BMI appears to be an independent factor associated with postoperative thoracic complications. Targeted perioperative strategies may reduce respiratory morbidity in high-BMI patients.

## Figures and Tables

**Table 1:** Baseline characteristics of the study population.

Characteristic	No, N = 37 ^1^	Yes, N = 10 ^1^	*p*-Value ^2^
Age	58 (51–65)	57 (50–63)	0.9
BMI	23.9 (22.8–27.3)	28.4 (26.4–29.3)	0.003
Menopause	31 (84%)	9 (90%)	>0.9
Charlson Comorbidity Index			0.4
CCI 0	18 (49%)	5 (50%)	
CCI 1	11 (30%)	1 (10%)	
CCI 2	7 (19%)	3 (30%)	
CCI 3	1 (2.7%)	1 (10%)	
Aletti’ Surgical Complexity Score	18.0 (15.0–21.0)	20.0 (14.0–21.0)	0.7
Operation Time	403 (321–460)	355 (279–438)	0.7
Estimated Blood Loss	500 (300–600)	550 (425–675)	0.3
ASA			0.033
2	20 (54%)	3 (30%)	
3	17 (46%)	5 (50%)	
4	0 (0%)	2 (20%)	
Intraoperative Drainage Placement	19 (51%)	4 (40%)	0.7
Type of Diagnosis			>0.9
Primary Diagnosis	35 (95%)	10 (100%)	
Recurrence	2 (5.4%)	0 (0%)	
Approach			0.7
Interval Debulking Surgery	19 (51%)	4 (40%)	
Primary Debulking Surgery	18 (49%)	6 (60%)	
Number of Neoadjuvant Chemotherapy cycles			0.8
3	2 (11%)	1 (25%)	
4	11 (61%)	3 (75%)	
5	1 (5.6%)	0 (0%)	
6	4 (22%)	0 (0%)	
Residual Tumor (cm)			>0.9
0	31 (84%)	9 (90%)	
0.5	1 (2.7%)	0 (0%)	
1	5 (14%)	1 (10%)	

^1^ Continuous variables are reported as median (IQR, 25th–75th percentile); categorical variables as n (%). ^2^ Wilcoxon rank sum test; Fisher’s exact test.

**Table 2:** Multivariate logistic regression analysis of predictors of thoracic complications.

Variable	Estimate	Std. Error	z Value	*p*-Value	Odds Ratio	95% CI
ASA	0.889	0.761	1.168	0.243	2.43	0.584–12.807
BMI	0.345	0.150	2.307	0.021	1.41	1.091–1.984

**Table 3:** Distribution of thoracic complications according to BMI categories.

Characteristic	Normal Weight, N = 25 ^1^	Over Weight, N = 18 ^1^	Obese, N = 4 ^1^	*p*-Value ^2^
Thoracic Complications	2, (8.0%)	6, (33%)	2, (50%)	0.033

^1^ n, (%); ^2^ Fisher’s exact test.

**Table 4:** Clinical characteristics of patients with thoracic complications.

Age	BMI	CCI	SCS	ASA	Type of Surgery	Complications
78	27.5	2	13	3	IDS	Bilateral Pleural Effusion
69	24.9	2	15	3	PDS	Bilateral Pleural Effusion
57	28	0	20	2	PDS	Unilateral Pleural Effusion, Bilateral Empyema
56	29.1	0	20	2	IDS	Unilateral Pleural Effusion
49	29.3	0	22	4	PDS	Unilateral Pleural Effusion
60	37.4	3	13	4	PDS	Pneumothorax, Pulmonary Embolism
64	28.7	1	21	3	IDS	Pneumothorax, Pulmonary Embolism
52	26.1	0	21	2	IDS	Unilateral Pleural Effusion
49	31.9	2	14	3	IDS	Unilateral Pleural Effusion
44	23.7	0	22	3	PDS	Unilateral Pleural Effusion

BMI—Body Mass Index Kg/m^2^; CCI—Charlson Comorbidity Index; SCS—Aletti’s Surgical Complexity Score; IDS—Interval Debulking Surgery; PDS—Primary Debulking Surgery.

## Data Availability

All data and the methodological process for their calculation can be supplied under explicit request to the corresponding author and provided as an ‘.R’ file. Data have been deposited on Zenodo at DOI 10.5281/zenodo.18431893.

## Abbreviations

The following abbreviations are used in this manuscript:

ASA	American Society of Anesthesiologists Physical Status Classification System
AUC	Area Under the Curve
BMI	Body Mass Index
CCI	Charlson Comorbidity Index
CI	Confidence Interval
HIPEC	Hyperthermic Intraperitoneal Chemotherapy
IDS	Interval Debulking Surgery
IRCCS	Istituto di Ricovero e Cura a Carattere Scientifico
OR	Odds Ratio
PDS	Primary Debulking Surgery
SD	Standard Deviation
SCS	Surgical Complexity Score (Aletti’s score)
STROBE	Strengthening the Reporting of Observational Studies in Epidemiology
